# Hypoallergenic and anti-inflammatory feeds in children with complicated severe acute malnutrition: an open randomised controlled 3-arm intervention trial in Malawi

**DOI:** 10.1038/s41598-019-38690-9

**Published:** 2019-02-19

**Authors:** Rosalie H. Bartels, Emmanuel Chimwezi, Victoria Watson, Leilei Pei, Isabel Potani, Benjamin Allubha, Kate Chidzalo, Duolao Wang, Queen Dube, Macpherson Mallewa, Angela Allen, Robert H. J. Bandsma, Wieger P. Voskuijl, Stephen J. Allen

**Affiliations:** 1 Global Child Health Group, Emma Children’s Hospital, Academic Medical Center, University of Amsterdam, Amsterdam, The Netherlands; 2 Department of Paediatrics and Child Health, Queen Elizabeth Central Hospital, Blantyre, Malawi; 3 The Childhood Acute Illness & Nutrition Network, Nairobi, Kenya; 4 Department of Clinical Sciences, Liverpool School of Tropical Medicine, Liverpool, UK; 5 College of Medicine, University of Malawi, Blantyre, Malawi; 6 Centre for Tropical Infectious Diseases, Liverpool School of Tropical Medicine, Liverpool, UK; 7 Division of Gastroenterology, Hepatology and Nutrition. The Hospital for Sick Children, Toronto, Ontario Canada; 8 Department of Nutritional Sciences, Faculty of Medicine, University of Toronto, Toronto, Ontario Canada; 9 Centre for Global Child Health, Hospital for Sick Children, Peter Gilgan Centre for Research & Learning, Toronto, Ontario Canada

## Abstract

Intestinal pathology in children with complicated severe acute malnutrition (SAM) persists despite standard management. Given the similarity with intestinal pathology in non-IgE mediated gastrointestinal food allergy and Crohn’s disease, we tested whether therapeutic feeds effective in treating these conditions may benefit children with complicated SAM. After initial clinical stabilisation, 95 children aged 6–23 months admitted at Queen Elizabeth Central Hospital, Blantyre, Malawi between January 1^st^ and December 31^st^, 2016 were allocated randomly to either standard feeds, an elemental feed or a polymeric feed for 14 days. Change in faecal calprotectin as a marker of intestinal inflammation and the primary outcome was similar in each arm: elemental vs. standard 4.1 μg/mg stool/day (95% CI, −29.9, 38.15; *P* = 0.81) and polymeric vs. standard 10 (−23.96, 43.91; *P* = 0.56). Biomarkers of intestinal and systemic inflammation and mucosal integrity were highly abnormal in most children at baseline and abnormal values persisted in all three arms. The enteropathy in complicated SAM did not respond to either standard feeds or alternative therapeutic feeds administered for up to 14 days. A better understanding of the pathogenesis of the gut pathology in complicated SAM is an urgent priority to inform the development of improved therapeutic interventions.

## Introduction

In 2016, severe wasting (weight-for-height Z score (WHZ) <−3) occurred in 17 million under-fives (2.5% of all children) and was estimated to account for between 7.4–7.8% of all child deaths^1–3^. A small percentage of children with severe acute malnutrition (SAM) require in-patient care because of poor feeding, medical complications such as severe oedema and infection, or failure to improve under community management^4^. Despite following a well-established World Health Organization (WHO) protocol^4^, case fatality in complicated SAM remains up to 35%^5–9^ with additional deaths after hospital discharge^8,10^.

Intestinal pathology in SAM is thought to result from increased exposure to microbial pathogens and poor nutrition^11–15^. Small intestinal biopsy studies in SAM revealed Th-1 dominated intestinal inflammation with variable degrees of villous atrophy, crypt hyperplasia and increased inflammatory infiltrate^11,16–21^. A recent biopsy and biomarker study in Zambian children with SAM and persistent diarrhoea not responding to standard therapy reported severe enteropathy with intestinal barrier failure and immune dysregulation^14^. These histological and immune features are similar to those that occur in children with Crohn’s disease^22^ and also non-IgE-mediated gastrointestinal food allergies (hereafter “food allergy”; e.g. due to cow’s milk protein) although the pathology of these latter disorders is poorly understood^20,21^. The similarities in the intestinal pathology in these conditions raises the intriguing possibility that treatments that reduce gut inflammation in Crohn’s disease and food allergy may also be of benefit in SAM and formed the rationale for evaluating the alternative therapeutic feeds in this study.

Intestinal inflammation in food allergy responds well to exclusion of the offending dietary antigen or, if the offending antigen is not known, a hypoallergenic, elemental feed composed of single amino acids^23^. In paediatric Crohn’s disease, first-line therapy is with exclusive enteral nutrition where all foods are replaced by an elemental formula or polymeric formula^22–25^. In limited previous research, hypoallergenic and elemental feeds were well tolerated in children with malnutrition and associated with improved weight gain^26,27^. However, assessment of impact in these studies was limited to nutritional recovery and did not assess effects on intestinal inflammation and other biomarkers of gut health. Also, recruitment in one study was limited to children who also had persistent diarrhoea^26^.

The concentration of calprotectin in faeces, a non-specific biomarker of intestinal inflammation, is validated in the diagnosis and management of inflammatory bowel disease^28^. Faecal calprotectin (FC) is markedly increased in SAM and remains elevated despite standard WHO management^12,19^.

Our hypothesis was that a hypoallergenic and an anti-inflammatory therapeutic formula would improve enteropathy and be well tolerated and safe in children with complicated SAM. The primary outcome was change in FC concentration. We also assessed biomarkers of intestinal integrity and systemic inflammation, tolerability of feeds, clinical outcomes and serious adverse events. Children managed with therapeutic feeds recommended by WHO formed the comparison group.

## Methods

### Study design

This randomised, open-label, 3-arm study was conducted at “Moyo” Nutritional Rehabilitation Unit, Queen Elizabeth Central Hospital, Blantyre, Malawi in accordance with the principles of good clinical practice and the Declaration of Helsinki^29^. The College of Medicine Research and Ethics Committee, Blantyre, Malawi (P.06/15/1745) and the Liverpool School of Tropical Medicine Ethics Committee, Liverpool, UK (15.048) gave approval.

### Participant recruitment

Children admitted from January 1^st^ to December 31^st^, 2016 were screened for eligibility. Inclusion criteria were age 6–23 months, SAM (WHZ <−3 and/or mid-upper arm circumference (MUAC) <11.5 cms and/or nutritional oedema)^30^; completed the stabilisation phase of management (assessed by their usual clinician to be clinically stable and tolerating F75 therapeutic feeds)^4^ and willing to stay on the ward for 2 weeks. We excluded children with a specific identifiable cause of malnutrition (e.g. feeding difficulties due to cerebral palsy; treatment for tuberculosis), those participating in another study or those with a sibling admitted with SAM. Both HIV positive and negative children were included. HIV positive children were already receiving ART before admission and this was continued during their care. Children were managed according to WHO guidelines using F75 therapeutic feeds during the stabilisation phase^4^.

Legal guardians were provided with verbal and written information either in Chichewa or English by a member of the study team. Following witnessed signed or thumbprint informed consent, research staff allocated children according to a computer-generated random sequence with blocks of random size in equal numbers to the three study arms. The legal guardian opened the next in a series of sealed, opaque envelopes each labelled with a unique study number and containing a coloured card indicating the intervention arm. Legal guardians were offered travel expenses.

### Interventions

Children were allocated to standard feeds (ready-to-use therapeutic food (RUTF) or, if not tolerated, F100), polymeric feed (Modulen IBD; Nestlé Health Science; York, UK) or elemental feed (PurAmino; Mead Johnson Nutrition; Chicago, 606 USA). Nutriset CMV (Complex of Minerals and Vitamins) (Malaunay, France) was added to both of the alternative feeds to approximate the nutrient content of F-100 (Supplementary Table S1). All feeds were introduced gradually over 2–3 days and provided 3-hourly for 14 days. Mothers were encouraged to re-establish or continue breastfeeding. No feeds other than breastfeeding and the intervention formulas were administered. Care was according to standard Malawian practice based on WHO guidelines which included a malaria slide, blood count and an HIV rapid antibody test, chloramphenicol and gentamicin as first line and ceftriaxone as second line antibiotic therapy and withholding oral iron until discharge^4^. All children received standard feeds after 14 days.

### Clinical data and biological sample collection

Demographic, anthropometric, socioeconomic and clinical information was collected at recruitment. Children were reviewed daily by the research team and volume of feed taken, symptoms reported by parents/caregivers, clinical signs and medications recorded. Research samples were collected alongside clinical samples whenever possible at recruitment, 7 and 14 days or within 48 hours of these time points. Stool samples were collected into sterile containers and transferred to a Calex tube. Up to 4 mls of venous blood was collected. 3 mls was transferred into EDTA anticoagulant and the remaining sample to a plain tube. The EDTA specimen was used to perform a full blood count by the hospital laboratory. The remaining EDTA sample and the plain tube were centrifuged and the plasma and serum, respectively, removed and stored at −80 °C at the Malawi Liverpool Wellcome Trust laboratory, Blantyre. Further processing and analyses were undertaken within the same laboratory.

### Biomarkers of systemic and intestinal inflammation, gut integrity and growth

Stool samples were analysed for calprotectin (Bühlmann fCAL ELISA; Schönenbuch, Switzerland) as a biomarker of intestinal inflammation. Faecal α_1_-antitrypsin (ELISA, Immunodiagnostik AG; Hamburg, Germany), plasma intestinal fatty acid binding protein (IFABP; Human FABP2/I-FABP Quantikine ELISA Kit; R&D Systems, Inc.; Minneapolis, USA) and plasma IgG anti-endotoxin antibody concentration (EndoCab Human, ELISA; Hycult biotech; Uden, The Netherlands) were measured as markers of intestinal mucosal integrity. Plasma C-reactive protein and α_1_-acid glycoprotein (Quantikine ELISA, R&D Systems, Inc.; Minneapolis, USA) were measured as markers of acute and chronic systemic inflammation respectively^31^. Plasma insulin like growth factor (IGF)-1 and IGF binding protein 3 (IGFBP3; Quantikine ELISA, R&D Systems, Inc.; Minneapolis, USA) as growth factors that are depressed in inflammation and SAM were also measured^14,32^. All analyses followed the manufacturer’s instructions. Normal ranges were those recommended by the manufacturers based on studies in healthy people. Laboratory staff was blinded to treatment allocations.

### Outcomes

The primary endpoint was the change in FC during the intervention period. Secondary outcomes were change in other biomarkers, gain in weight (g/kg/day after resolution of oedema if present), diarrhoea reported by caregivers and tolerance of feeds (vomiting reported by caregivers and requirement for naso-gastric tube feeds). Suspected sepsis was defined as clinical suspicion and the start or change in antibiotic therapy by the child’s clinical team. Treatment-emergent serious adverse events (SAEs), defined as events that commenced, or worsened, after the allocated feed had been administered, were reviewed by a senior paediatrician and reported to the Pharmacovigilance Pharmacist at LSTM. SAEs were reviewed by two blinded independent safety monitors and categorised according to Medical Dictionary for Regulatory Activities (MedDRA) Preferred term.

### Participant withdrawal

Legal guardians were free to withdraw their child at any time. Participants were asked to provide an additional stool and blood sample on withdrawal from the study.

### Sample size calculation

The geometric mean (standard deviation (SD)) for FC at recruitment in 37 malnourished Kenyan children was 2.69 (0.45); (K Jones, personal communication)^19^. At the 5% significance level and with a coefficient of variation of 0.4, a total of 90 children (30 in each group) was needed to detect a 25% reduction in geometric mean calprotectin in each of the alternative feed arms compared with the standard arm with at least 81% power. We intended to recruit 120 children (40/group) to allow for deaths and dropouts.

### Statistical analysis

The effects of the therapeutic feeds were assessed according to average change from baseline in laboratory and clinical variables. The earliest and latest stool and blood samples available during the 14-day intervention period were used to assess change in biomarkers. The primary endpoint was analysed using a generalised linear model (GLM) with treatment as predictor and baseline FC as covariate. The model had Gaussian distribution and identity link function. The treatment difference in the mean of the primary outcome and its 95% CI were derived from the GLM model. Other biomarkers and clinical endpoints were analysed similarly with distributions and link functions determined by type of data (continuous, binary and count). For repeated measurement outcomes, mixed models with treatment as fixed effect, baseline measurement as covariate, and subject as random effect were used to derive treatment differences and 95% CIs. The primary endpoint analysis was based on the intention to treat (ITT) population. Additional analysis in the ITT and per protocol population of the primary and secondary endpoints were also performed. Also, data were also analysed using a t-test to detect any differences in laboratory and clinical parameters between alternative feed and standard arms at baseline (up to day 3) and day 14 (±3 days). No imputation was made for the outcome or baseline variables. No interim analysis was conducted. Analysis was performed using SAS v9.4.

## Results

**Figure 1:**
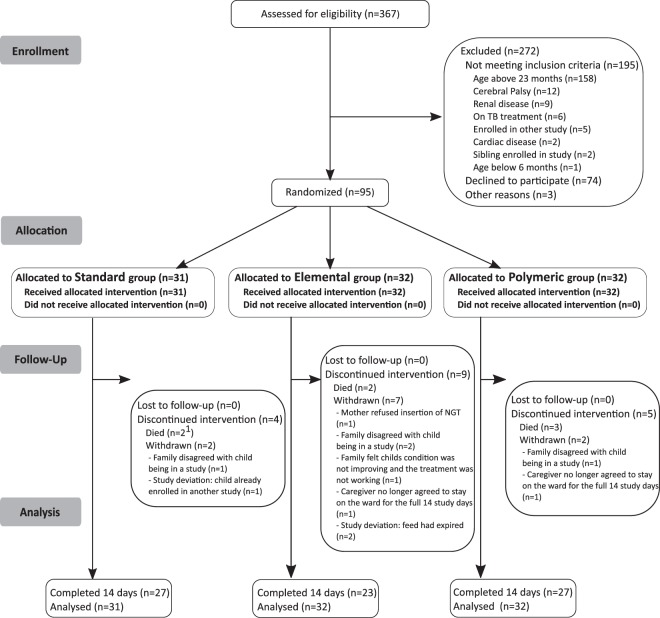
Flowchart. Study flow diagram of children with severe acute malnutrition assessed for eligibility and recruited for the study. ^1^Two additional children were known to have died after the 14-day study period and are not included in the Figure.

Three hundred and sixty-seven children admitted with complicated SAM were screened. Of the 172 (46.9%) eligible children, 95 (55.2%) were randomly assigned to the three treatment arms (Fig. 1). Excluding study deviations, the number of children withdrawn by families was similar in the standard (3 children), elemental (5 children) and polymeric arms (3; *P* = 0.31). Withdrawals were mostly related to family concerns with the child being enrolled in a research study and refusal to remain under admission.

Baseline demographic and clinical characteristics (Table 1) and socioeconomic factors (Supplementary Table S2) were similar in the three arms. Mean (SD) age was 15.6 (5.7) months and most children lived in either an urban or peri-urban setting. Thirty-five (36.8%) children were breastfed and all but one child was receiving complementary feeds. Mean (SD) MUAC was 10.7 cm (1.2) and WHZ in children without oedema was −3.74 (1.25). Stunting was common (height-for-age z score −2.94 (2.06)). Thirty-eight (40.0%) children with oedematous malnutrition were older (*P* < 0.001) and had a greater mean MUAC (P < 0.001) than those without oedema (Supplementary Table S3). Thirty-four (36.2%) children positive for HIV had a lower mean MUAC than HIV negative children (*P* = 0.002; Supplementary Table S4). Children in the standard arm all received RUTF; only 6 required some F-100 during their care.

**Table 1:** Baseline demographic and clinical characteristics according to intervention arm.

Variable^a^	Standard N = 31	Elemental N = 32	Polymeric N = 32	Total N = 95
**Demographic variables**				
Age in months (mean; SD)	16.6 (5.4)	14.8 (6.6)	15.5 (5.2)	15.6 (5.74)
Male	14 (45.2)	15 (46.9)	17 (53.1)	46 (48.4)
Residence type				
Rural	10 (32.3)	9 (28.1)	10 (31.3)	29 (30.5)
Urban	14 (45.2)	19 (59.4)	17 (53.1)	50 (52.6)
Peri-urban	7 (22.6)	4 (12.5)	5 (15.6)	16 (16.8)
Mother main caregiver	30 (96.8)	29 (90.6)	27 (84.4)	86 (90.5)
Mother HIV positive	10 (37)	14 (43.8)	14 (43.8)	38 (40)
Mother died	1 (3.2)	1 (3.1)	2 (6.3)	4 (4.2)
Number of siblings alive (mean; SD)	1.87 (1.5)	1.78 (1.5)	1.13 (1.3)	1.59 (1.45)
Number of siblings died (mean; SD)	0.19 (0.5)	0.13 (0.42)	0.41 (1.0)	0.24 (0.70)
**Clinical variables**				
Child HIV positive	11 (35.5)	12 (37.5)	11 (34.4)^b^	34 (35.8)
Breastfed	22 (71)	20 (62.5)	20 (62.5)	62 (65.3)
Receiving complementary feeds	31 (100)	31 (96.9)	32 (100)	94 (98.9)
Oedema				
None	17 (54.8)	18 (56.3)	22 (68.8)	57 (60)
+	4 (12.9)	3 (9.38)	3 (9.38)	10 (10.5)
++	5 (16.1)	9 (28.1)	6 (18.8)	20 (21.1)
+++	5 (16.1)	2 (6.25)	1 (3.13)	8 (8.42)
Mid upper arm circumference (cms; mean; SD)^c^	10.3 (1.2)	10.5 (1.27)	11.3 (0.81)	10.7 (1.2)
Weight-for-height z score (mean; SD)^d^	−4.10 (1.34)	−3.79 (1.28)	−3.41 (1.13)	−3.74 (1.25)
Length-for-age z score (mean; SD)	−2.80 (1.49)	−3.01 (2.85)	−3.00 (1.60)	−2.94 (2.06)

^a^Values are number (%) unless otherwise stated; ^b^HIV status not known for 1 child; ^c^Only reported for children without severe oedema (++, +++); ^d^Only reported for children without oedema.

### Biomarkers

**Figure 2:**
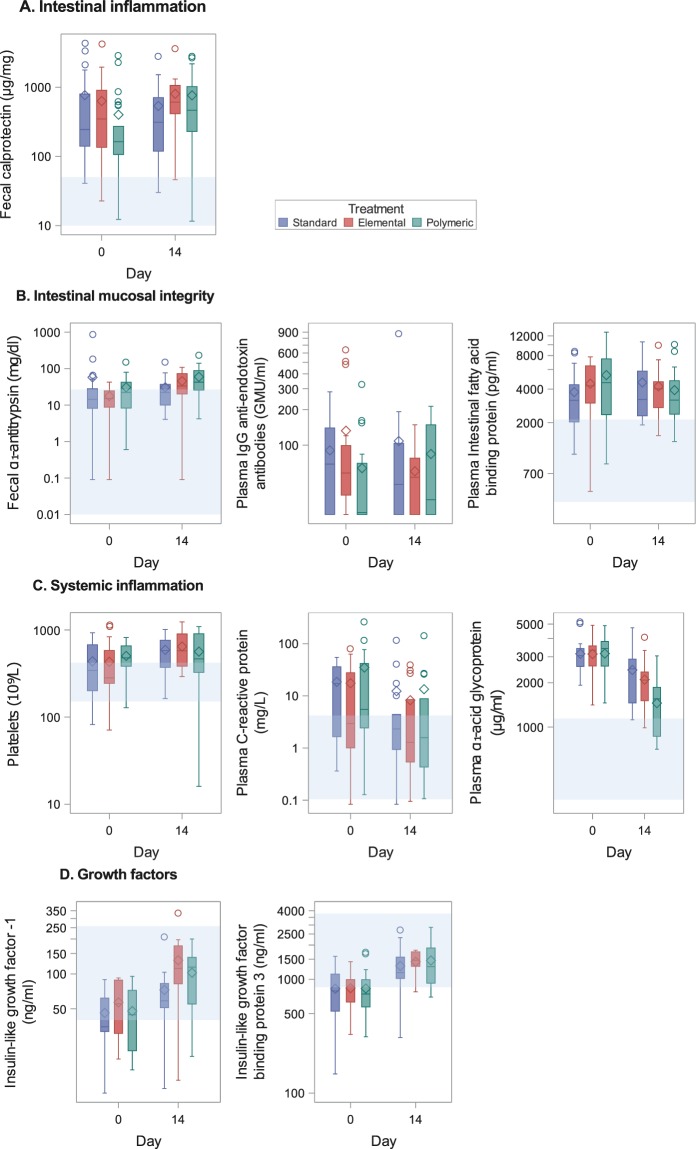
Biomarkers according to intervention arm. Box plots show values at baseline (or up to day 3) and day 14 (±3 days) according to intervention group (standard: blue, elemental: red, polymeric: green). Boxplots summarise the median (midline), the mean (diamond marker) and interquartile ranges (upper and lower box). Whiskers are drawn to the nearest value within 1.5 times the IQR. Values outside of this range are shown as circles. Light blue shading shows normal range.

Geometric mean values for biomarkers at baseline (within 3 days of recruitment) and day 14 (±3 days) according to intervention arm are shown in Fig. 2 and Supplementary Table S5. Change in biomarkers in the intervention arms versus the standard arm is shown in Table 2. At baseline, the gut, systemic inflammation and growth biomarkers were abnormal in most children with similar values in the intervention and standard arms (Fig. 2 and Supplementary Table S5).

**Table 2:** Change in laboratory biomarkers and clinical variables according to intervention arm^a^.

Variable	Elemental vs Standard			Polymeric vs Standard		
	N^b^	Change/day (95% CI)	*P* value	N^c^	Change/day (95% CI)	*P* value
**Intestinal inflammation**						
Faecal calprotectin (μg/g stool)	26/25	4.1 (−29.9, 38.2)	0.81	26/25	10 (−24.0, 43.9)	0.56
**Intestinal integrity**						
Faecal α_1_-antitrypsin (mg/dl)	21/23	1.7 (−0.42, 3.8)	0.12	22/23	3.5 (1.4, 5.5)	0.0013
Plasma IgG anti-endotoxin antibodies (GMU/ml)	16/18	−1.8 (−11.8, 8.3)	0.72	17/18	−2.1 (−11.7, 7.5)	0.66
Plasma Intestinal fatty acid binding protein (pg/ml)	16/17	−194.3 (−387.6, −1.0)	0.05	17/17	−83.8 (−276.2, 108.5)	0.38
**Systemic inflammation**						
Platelets (x10^9^/L blood)	15/19	7.4 (−9.1, 23.9)	0.37	17/19	−7.1 (−23.0, 8.9)	0.38
Plasma C-reactive protein (mg/L)	16/17	−0.7 (−2.2, 0.81)	0.35	17/17	−0.04 (−1.55, 1 0.46)	0.95
Plasma α_1_-acid glycoprotein (μg/ml)	16/16	−15.7 (−84.8, 53.4)	0.65	17/16	−89.4 (−157.7, 21.1)	0.01
**Growth Factors**						
Insulin-like growth factor −1 (ng/ml)	9/9	4.4 (−2.1, 10.9)	0.17	14/9	4.3 (−1.3, 9.9)	0.13
Insulin-like growth factor binding; protein 3 (ng/ml)	16/17	22.3 (−29.7, 74.4)	0.39	17/17	37.1 (−14.1, 88.3)	0.15
**Clinical**						
Weight (g/kg/day)	30/30	1.6 (−3.6, 6.8)	0.54	31/30	2.5 (−2.1, 7.7)	0.33
MUAC (cms/day)	31/30	0.025 (−0.025, 0.07)	0.32	32/30	0.042 (−0.009, 0.09)	0.1
Weight-for-length z-score	30/30	0.22 (−0.29, 0.76)	0.39	31/30	0.19 (−0.31, 0.7)	0.45

^a^Data are number of children and generalised linear analysis of mean (95% CI) difference in change in variable/day in the alternative feed compared to standard feed. A negative value signifies a fall in the variable with the alternative therapeutic feed compared with standard feeds and *vice versa* for a positive value. Laboratory measurements were made in the first and last samples available and clinical measurements on recruitment and the last measurement available during the 14-day intervention period.

^b^Numbers represent the data available for the elemental/standard treatment group.

^c^Numbers represent the data available for the polymeric/standard treatment group.

MUAC, mid-upper arm circumference.

Mean FC was markedly elevated at baseline (mean (SD) 547 (744) μg/g stool; normal <50) and remained elevated at 14 days (697 (735); *P* = 0.31; Supplementary Table S5). Change in FC was similar in the intervention versus standard arms: elemental vs. standard 4.1 μg/mg stool/day (95% CI, −29.9, 38.15; *P* = 0.81) and polymeric vs. standard 10.0 (−23.96, 43.91; *P* = 0.56; Table 2).

Change in mucosal integrity biomarkers were generally similar in the intervention versus the standard arms. The exceptions were faecal α_1_-antitrypsin which increased significantly during treatment (*P* = 0.0046) and to a greater extent in the polymeric versus the standard arm (*P* = 0.0013) and IFABP which fell in the elemental compared with the standard arm (*P* = 0.049).

Change in systemic inflammation biomarkers was also generally similar in the intervention versus the standard arms. The exception was plasma α_1_-acid glycoprotein which fell significantly in all children (*P* < 0.0001) and to a greater extent in the polymeric compared with the standard arm (*P* = 0.01).

IGF-1 and IGFBP3 levels were low at recruitment and increased significantly during treatment (*P* = < 0.0001 for both) and to a similar degree in the intervention and standard arms. Tracking changes in biomarkers in individual children did not identify subgroups of children who responded either better or worse to the interventions (Supplementary Fig. S1).

Children with oedema had a significantly higher mean plasma α_1_-acid glycoprotein at recruitment than those without (*P* = 0.024; Supplementary Table S3). HIV positive children had higher mean baseline values for FC (*P* = 0.005) and plasma C-reactive protein (*P* = 0.008) and lower haemoglobin (*P* = 0.007) than HIV negative children (Supplementary Table S4).

### Nutritional recovery and clinical outcomes

**Figure 3:**
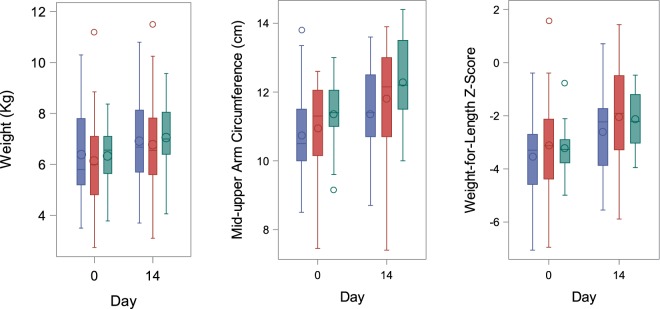
Anthropometry according to intervention arm. Box plots show parameter at baseline (or up to day 3) and day 14 (±3 days) according to intervention group (standard: blue, elemental: red, polymeric: green) and show the median (midline), the mean (diamond marker) and interquartile ranges (upper and lower box). Whiskers are drawn to the nearest value within 1.5 times the IQR. Values outside of this range are shown as circles.

Weight gain and change in MUAC and weight-for-length z score were similar in the intervention arms versus the standard arm (Table 2; Fig. 3). The number of days to resolution of oedema was similar in the standard, elemental and polymeric arms (mean (SD): 2.38 (1.12); 2.42 (1.44); 2.0 (1.12) respectively). Loose or watery stools were very common in all three arms (Supplementary Table S6). The alternative feeds were tolerated less well than standard feeds (mostly RUTF) with greater requirement for nasogastric tube (NGT) feeding and caregiver reporting of vomiting. In addition, amongst those affected, vomiting occurred more frequently with the elemental compared with standard feeds (average difference = 2.22/day, 95% CI = 1.08, 4.58; *P* = 0.031). However, reverting back to F75 was not required with the polymeric feed (Supplementary Table S6). The number of days with loose stools and vomiting amongst children who experienced these symptoms was similar in each arm.

When HIV status or oedema at recruitment were included in the GLM, there was no evidence of interaction between treatment arm and changes in clinical or laboratory outcomes except that plasma α_1_-acid glycoprotein fell to greater degree in children with oedema than those without (*P* = 0.025).

### Serious adverse events and deaths

A total of 43 SAEs occurred in 25 (27.4%) children with a similar frequency in each arm (*P* = 0.5; Supplementary Table S7). The most frequent SAEs were gastroenteritis (13.7% children), dehydration (11.6%) and sepsis (6.3%). Gastroenteritis and dehydration in two children and an urticarial rash in one child were considered possibly related to the therapeutic feeds and all occurred in the standard arm. Seven children died (7.4%) within the 14-day study period; 5 deaths (71%) occurred in HIV positive children and 3 (43%) in children with oedema. Another two children died on day 15 with death attributed to a SAE that started within the study period; both of these children were in the standard arm. The number of deaths was similar in each intervention arm (Supplementary Table S7).

## Discussion

This study confirms previous observations that compromised gut integrity and intestinal and systemic inflammation occur in complicated SAM. Despite moderate weight gain and a rise in growth factors in all three arms, biomarkers of intestinal pathology did not improve during 14 days treatment either with standard or the alternative therapeutic feeds. The biomarker findings are consistent with the on-going or recurrent diarrhoea in many children and the persistence of histological abnormalities despite treatment reported previously^16^. The persistence of the gut pathology likely contributes to the 10–30% case-fatality^6,12,33–37^, with even higher mortality in HIV positive children^8,38^, and also poor longer-term outcomes with mortality after hospital discharge typically around 25%^8,10^.

The degree of intestinal inflammation in our children with complicated SAM based on FC concentrations are similar to that observed in Crohn’s disease^28^. Severely impaired mucosal integrity in our children was evidenced by marked abnormalities in three biomarkers. Levels of plasma I-FABP, reflecting enterocyte destruction^39^, were similar to those in south African children with SAM^40^, active Crohn’s disease^41^ and greater than those reported in adults with acute mesenteric ischemia^42,43^. Faecal α_1_-antitrypsin, a biomarker of intestinal protein loss and moderately increased in our patients, is also elevated in Crohn’s disease^44^. Finally, anti-endotoxin antibodies in plasma likely indicates translocation of gut bacteria^40^.

Significant systemic inflammation at recruitment was evidenced by raised CRP, platelet count and plasma α_1_-acid glycoprotein consistent with findings in inflammatory bowel disease^45^. Although plasma α_1_-acid glycoprotein, a biomarker of chronic inflammation, fell significantly during treatment, platelet counts increased and abnormal levels for all three biomarkers persisted in most children. In complicated SAM, systemic inflammation may result from infections, including translocation of gut bacteria^40^, as well as intestinal inflammation^12^. The fall in plasma α_1_-acid glycoprotein may reflect treatment of systemic infection rather than reduced intestinal inflammation.

The failure of the two alternative feeds to improve intestinal pathology, systemic inflammation or clinical outcomes contrasts markedly with their clinical efficacy in non-IgE mediated food allergy^20,21^ and Crohn’s disease. In the latter, although the mechanism is unknown, exclusive enteral nutrition with either a polymeric or elemental formula reduces intestinal inflammation, promotes mucosal healing, down regulates pro-inflammatory cytokines and improves nutritional status^16–19^. Although we observed a greater fall in plasma α_1_-acid glycoprotein in the polymeric arm, possibly related to its content of transforming growth factor beta^46^, in the absence of a similar effect on other biomarkers of systemic inflammation and an increase in faecal *α*_1_-antitrypsin with the polymeric feed, the clinical relevance of this finding is unclear. The greater fall in IFABP in the elemental compared with the standard group was of borderline statistical significance and, in the absence of associated clinical benefits, is unlikely to be of clinical significance. The lack of efficacy of the elemental formula indicates that exclusion of dietary antigens present in either cow milk based therapeutic feeds or RUTF is not effective in improving enteropathy. The discrepancy between our findings with older studies of elemental formula which reported improved weight gain in malnourished children with diarrhoea^26,27^ may be due to differences in the patient population or the use of RUTF as standard treatment in our study.

The higher *α*_1_-acid glycoprotein at recruitment in oedematous children may reflect greater bacterial translocation consistent with impaired intestinal integrity related to deficient glycosylation reported in kwashiorkor^17^. However, anti-endotoxin antibodies and other markers of gut integrity did not differ significantly according to presence of oedema. CRP and FC were particularly elevated in HIV positive children at recruitment, the latter consistent with findings in HIV infected Zambian children with SAM and persistent diarrhoea^14^. However, biomarkers of mucosal integrity did not differ significantly according to HIV status. This has also been reported in South African children with SAM in whom plasma IFABP and also plasma 16sDNA as a marker of microbial translocation were similar in those with and without HIV^40^. The contribution of HIV to the enteropathy in complicated SAM requires further study.

Our study had several limitations. We recruited only 55% of eligible children which limits the generalisability of our findings. Sample collection was difficult in these sick children who are already having samples collected for clinical purposes. This limited the number of samples available for the measurement of several of the biomarkers and risks a false negative result that incorrectly rejects a useful intervention. However, the findings across the clinical and laboratory variables did not suggest a trend towards a beneficial effect of either of the two intervention feeds compared with standard feeds. In this open study, reporting of symptoms and clinical observations could have been biased. However, measurement of biomarkers was blinded and these findings were consistent with the non-blinded observations. Finally, although exclusive enteral nutrition with a polymeric formula in Crohn’s disease reduced systemic inflammation within 3–14 days^47–49^, and guidelines recommend that an alternative therapy should be considered if a clinical response has not occurred within 2 weeks^50^, it is possible that feeding for longer than 14 days may have improved outcomes in our cases. However, in our setting, longer periods of exclusive enteral nutrition would be impractical given their poorer tolerance than RUTF, greater requirement for NGT feeding and likely need for prolonged hospital admission to supervise feeding which also carries the risk of increased exposure to hospital acquired infections.

We are not aware of any previous studies of interventions for enteropathy in children with complicated SAM. In a previous study on the Moyo ward, a synbiotic supplement did not improve clinical outcomes^51^.

Our findings that neither the elemental nor polymeric feeds administered for up to 14 days resulted in any clear improvement in biomarkers of intestinal inflammation and integrity, systemic inflammation or clinical benefit in complicated SAM suggests multifactorial intestinal pathology. The enteropathy in complicated SAM may be a continuum of environmental enteric dysfunction (EED) that is ubiquitous in unhygienic settings^13^. Recent studies of EED have highlighted the critical importance of sub-clinical infection with enteropathogens in compromising mucosal integrity and causing intestinal and systemic inflammation^52^ and the high burden of gut pathogens in complicated SAM was associated with intestinal inflammation^12^. Targeting enteropathogens in complicated SAM may be required to improve the enteropathy.

## Conclusions

Current WHO management fails to improve intestinal pathology in complicated SAM. Elemental and polymeric feeds did not have the hoped anti-inflammatory or clinical benefits and proved to be poorly tolerated. Further research is needed to better understand the intestinal pathology in complicated SAM to help develop interventions that may address the unacceptably high case-fatality and poor long-term outcomes.

## Supplementary information

Supplementary Figure 1

Supplementary Tables S1-S7

## Data Availability

The datasets generated during this study will be available from the corresponding author on reasonable request.
