# Supplementary material for: Hypoallergenic and anti-inflammatory feeds in children with complicated severe acute malnutrition: an open randomised controlled 3-arm intervention trial in Malawi
Source: Sci Rep. 2019 Feb 19;9:2304. doi: 10.1038/s41598-019-38690-9 (PMC6381085; doi:10.1038/s41598-019-38690-9)
Supplement: Supplementary file 1 — Supplementary Figure 1 [file 41598_2019_38690_MOESM1_ESM.docx]

**TITLE PAGE**

1. **Title**

**Hypoallergenic and anti-inflammatory feeds in children with complicated severe acute malnutrition: an open randomised controlled 3-arm intervention trial in Malawi**

1. **Author Names**

Rosalie H. Bartels (PhD)^1,2*^, Emmanuel Chimwezi (BSc)^2,3^, Victoria Watson (BSc)^4^, Leilei Pei (PhD)^4^, Isabel Potani (MSc)^2^, Benjamin Allubha (BSc)^2^, Kate Chidzalo (BSc)^2,3^, Duolao Wang (PhD)^4^, Queen Dube (PhD)^5^, Macpherson Mallewa (PhD)^5^, Angela Allen (PhD)^6^, Robert H.J. Bandsma (PhD)^3,7,8,9^, Wieger P. Voskuijl (PhD)^1,2,3^, Stephen J Allen (MD)^4^

**Supplementary Figure S1** – **Changes in biomarkers in individual children according to intervention arm**


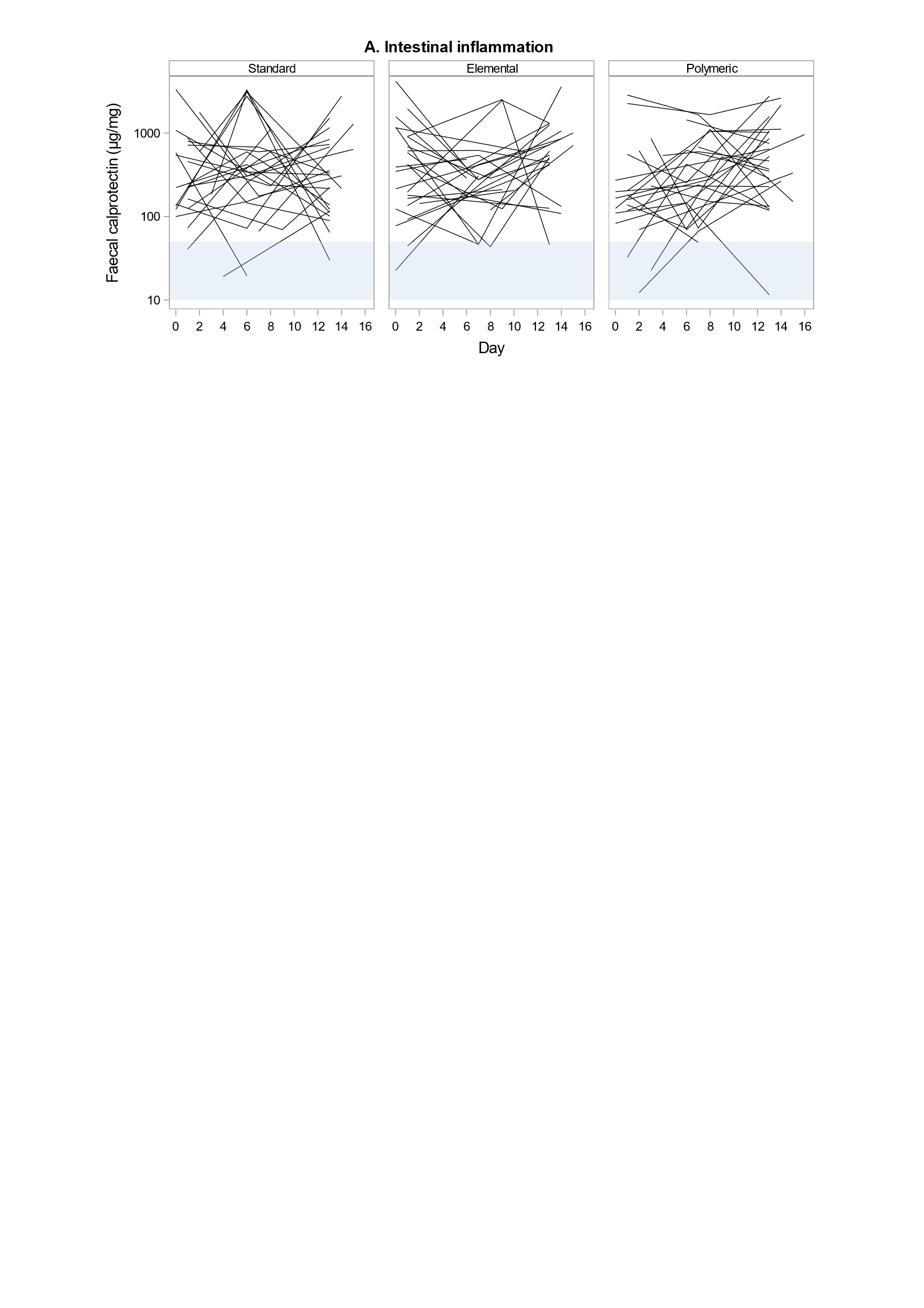


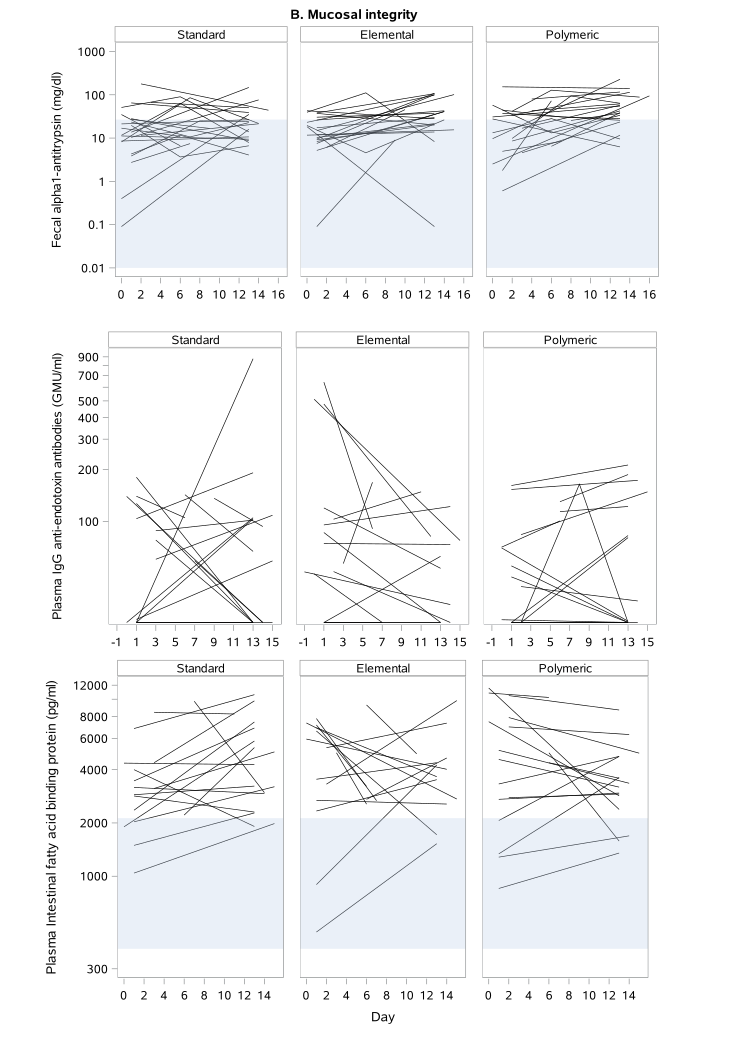

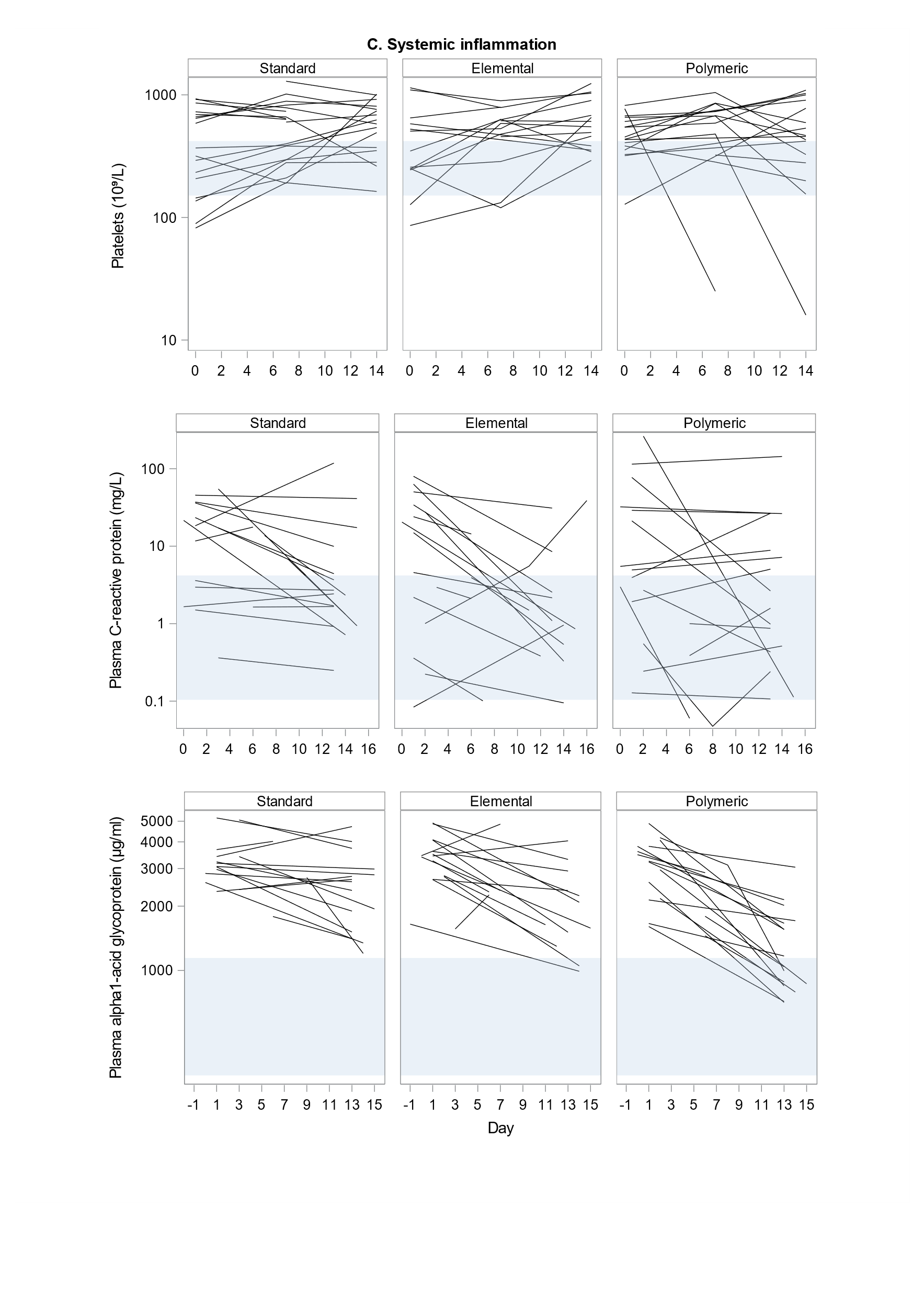

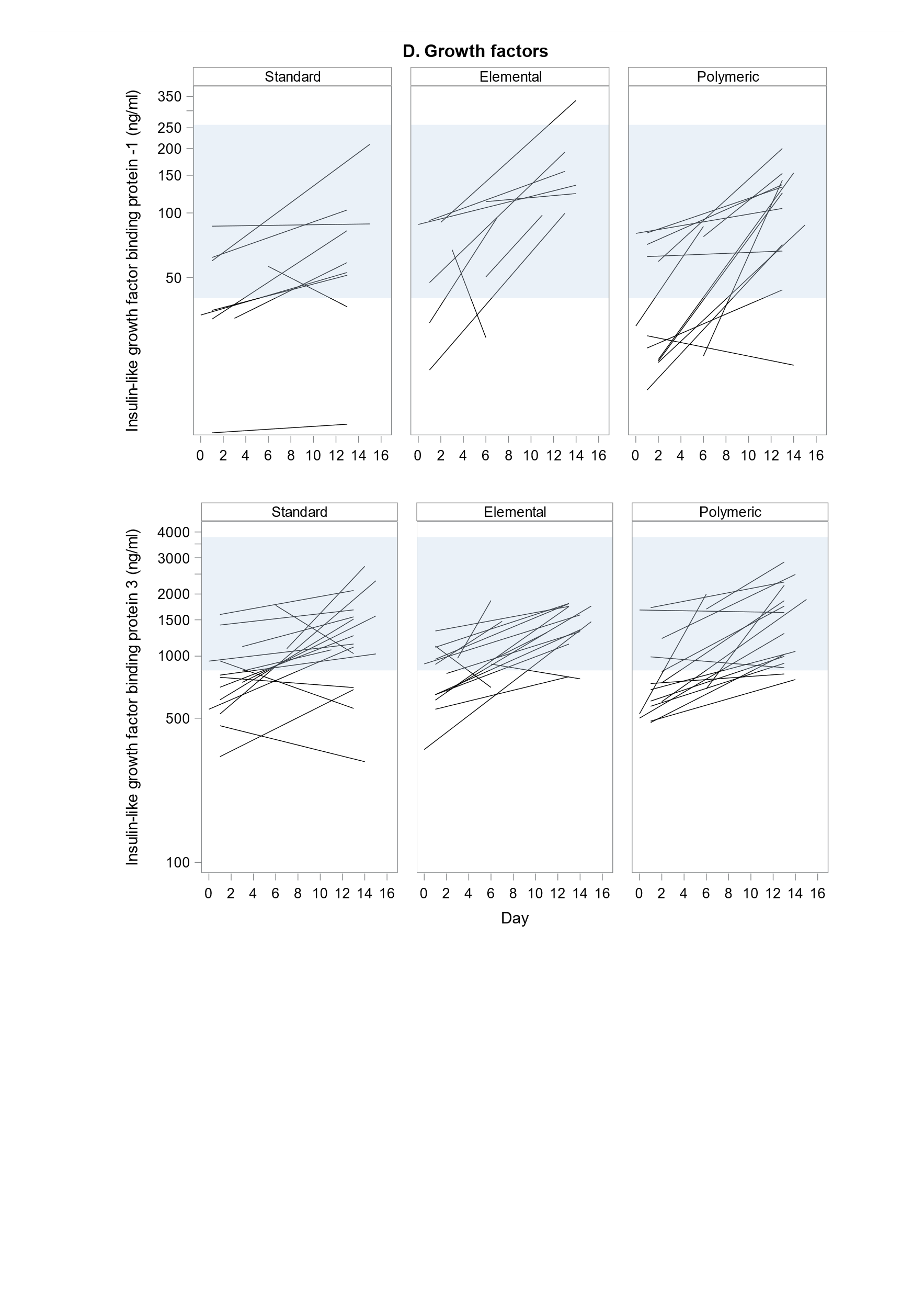


Light blue shading shows normal range.
