# Supplementary material for: Hypoallergenic and anti-inflammatory feeds in children with complicated severe acute malnutrition: an open randomised controlled 3-arm intervention trial in Malawi
Source: Sci Rep. 2019 Feb 19;9:2304. doi: 10.1038/s41598-019-38690-9 (PMC6381085; doi:10.1038/s41598-019-38690-9)
Supplement: Supplementary file 2 — Supplementary Tables S1-S7 [file 41598_2019_38690_MOESM2_ESM.docx]

**TITLE PAGE**

1. **Title**

**Hypoallergenic and anti-inflammatory feeds in children with complicated severe acute malnutrition: an open randomised controlled 3-arm intervention trial in Malawi**

1. **Author Names**

Rosalie H. Bartels (PhD)^1,2*^, Emmanuel Chimwezi (BSc)^2,3^, Victoria Watson (BSc)^4^, Leilei Pei (PhD)^4^, Isabel Potani (MSc)^2^, Benjamin Allubha (BSc)^2^, Kate Chidzalo (BSc)^2,3^, Duolao Wang (PhD)^4^, Queen Dube (PhD)^5^, Macpherson Mallewa (PhD)^5^, Angela Allen (PhD)^6^, Robert H.J. Bandsma (PhD)^3,7,8,9^, Wieger P. Voskuijl (PhD)^1,2,3^, Stephen J Allen (MD)^4^

Supplementary Table S1 – **Composition of therapeutic feeds per litre**

| **Nutrient** | **RUTF**  **(Per sachet of 92g)** | **F-100^1^** | **Modulen IBD^2,3^ with 1.7g CMV/L** | **PurAmino^2,4,5^ with 1.7g CMV/L** |
| --- | --- | --- | --- | --- |
| Energy (kcal) | 500 | 998 | 1000 | 1000 |
| Protein (g) | 12.5 | 29 | 36 | 27.8 |
| Carbohydrates (g) |  | 50 | 110 | 105.9 |
| Fat (g) | 32.86 | 59 | 47 | 52.9 |
| Osmolality (mOsmol/l) |  | 419 | 290 | 450.6 |
| **Minerals** |  |  |  |  |
| Sodium (mg) | < 267 | 560 | 350.0 | 470.6 |
| Potassium (mg) | 1022 | 2300 | 1782.3 | 1670.5 |
| Calcium (mg) | 276 | 850 | 910.0 | 941.2 |
| Phosphorus (mg) | 276 | 825 | 610.0 | 514.7 |
| Magnesium (mg) | 84.6 | 154 | 263.3 | 172.1 |
| Zinc (mg) | 12.9 | 21.2 | 21.6 | 22.0 |
| Copper (mg) | 1.6 | 3 | 2.5 | 2.3 |
| Iron (mg) | 10.6 | 0.4 | 11.0 | 17.9 |
| Manganese (mg) | - | - | 2 | - |
| Fluoride (μg) | - | - | <20 | - |
| Chromium (μg) | - | - | 51 | - |
| Molybdenum (μg) | - | - | 75 | - |
| Selenium (μg) | 27.6 | 57 | 35.0 | 27.8 |
| Iodine (μg) | 92 | 225 | 100.1 | 148.6 |
| **Vitamins** |  |  |  |  |
| A μg RE | 840 | 1544 | 1911.0 | 1968.1 |
| D μg | 15 | 29 | 27.0 | 29.5 |
| E mg **α**-TE | 18.4 | 38.6 | 32.4 | 32.8 |
| K μg | 19.3 | 29 | 76.3 | 111.0 |
| C mg | 49 | 96.5 | 145.5 | 167.6 |
| B1 (Thiamin) mg | 0.55 | 0.97 | 1.7 | 1.3 |
| B2 (Riboflavin) mg | 1.66 | 3.1 | 2.8 | 2.4 |
| Pantothenic acid mg | 2.85 | 5.8 | 8.0 | 8.0 |
| B6 mg | 0.55 | 1.2 | 2.3 | 1.2 |
| B12 μg | 1.7 | 3.1 | 4.7 | 4.5 |
| Niacin mg | 4.88 | 9.7 | 16.8 | 14.8 |
| Folic acid μg | 193 | 386 | 435.5 | 354.3 |
| Biotin μg | 60 | 116 | 89.8 | 87.2 |

1. For composition, see: <http://apps.who.int/iris/bitstream/10665/41999/1/a57361.pdf>

Both feeds were prepared by adding cooled, boiled water to 400g to make-up to 2L and then adding 1.7g CMV (Complex of Minerals and Vitamins)/L

1. For composition, see: <https://www.nestlehealthscience.co.uk/asset-library/documents/data%20card%20modulen%20ibd.pdf>
2. For composition, see: <http://www.nutramigen.co.uk/files/5114/2565/5560/Filofax_Puramino_01_2015_5.pdf>
3. Prepared as described results in a formula with approx. 1kcal/ml (<http://www.meadjohnson.com/pediatrics/us-en/sites/hcp-usa/files/345%20PurAmino%20Scoop%20Dilution.pdf>)

RUTF, ready-to-use therapeutic food.

Supplementary Table S2 – **Socioeconomic variables at recruitment** **according to intervention arm**

| **Variable N (%)** | **Standard**  **(N=31)** | **Elemental (N=32)** | **Polymeric (N=32)** | **Total (N=95)** |
| --- | --- | --- | --- | --- |
| House type |  |  |  |  |
| • Owned | 12 (38.7) | 19 (59.4) | 13 (40.6) | 44 (46.3) |
| • Rented | 19 (61.3) | 12 (37.5) | 18 (56.3) | 49 (51.6) |
| • Other | 0 (0.0) | 1 (3.13) | 1 (3.13) | 2 (2.11) |
| Number sleeping rooms (mean; SD) | 1.97 (0.71) | 2.56 (1.29) | 2.13 (0.91) | 2.22 (1.02) |
| Number of people usually sleeping in the house (mean; SD) | 4.61 (1.84) | 4.66 (1.77) | 4.81 (2.05) | 4.69 (1.87) |
| Electricity supply | 8 (25.8) | 10 (31.3) | 5 (15.6) | 23 (24.2) |
| The family owns: |  |  |  |  |
| • A radio | 11 (35.5) | 16 (50) | 10 (31.3) | 37 (38.9) |
| • A bicycle | 2 (6.45) | 5 (15.6) | 4 (12.5) | 11 (11.6) |
| • A motorbike | 0 (0.0) | 1 (3.13) | 1 (3.13) | 2 (2.11) |
| • A car or truck | 1 (3.23) | 0 (0.0) | 0 (0.0) | 1 (1.05) |
| • A paraffin lamp | 7 (22.6) | 10 (31.3) | 8 (25) | 25 (26.3) |
| • A koloboyi (paraffin lamp) | 10 (32.3) | 9 (28.1) | 18 (56.3) | 37 (38.9) |
| • An oxcart | 0 (0.0) | 1 (3.13) | 0 (0.0) | 1 (1.05) |
| • A television | 4 (12.9) | 10 (31.3) | 3 (9.38) | 17 (17.9) |
| • A cell phone | 12 (38.7) | 14 (43.8) | 15 (46.9) | 41 (43.2) |
| • A telephone (landline) | 0 (0.0) | 1 (3.13) | 1 (3.13) | 2 (2.11) |
| • A bed with mattress | 12 (38.7) | 20 (62.5) | 7 (21.9) | 39 (41.1) |
| • A sofa set | 4 (12.9) | 10 (31.3) | 3 (9.38) | 17 (17.9) |
| • A table and chair | 16 (51.6) | 23 (71.9) | 16 (50) | 55 (57.9) |
| • A refrigerator | 2 (6.45) | 4 (12.5) | 2 (6.25) | 8 (8.42) |
| • A watch | 5 (16.1) | 10 (31.3) | 4 (12.5) | 19 (20) |
| The family owns agricultural land |  |  |  |  |
|  | 21 (67.7) | 25 (78.1) | 19 (59.4) | 65 (68.4) |
| The principal households source of drinking water is (N; %): |  |  |  |  |
| • Pipe inside dwelling | 1 (3.23) | 0 (0.0) | 0 (0.0) | 1 (1.05) |
| • Pipe outside dwelling/ to yard | 1 (3.23) | 3 (9.38) | 1 (3.13) | 5 (5.26) |
| • Protected borehole/ well | 9 (29) | 15 (46.9) | 9 (28.1) | 33 (34.7) |
| • Public tap | 16 (51.6) | 11 (34.4) | 18 (56.3) | 45 (47.4) |
| • Traditional public well | 4 (12.9) | 3 (9.38) | 4 (12.5) | 11 (11.6) |
| • River/ canal/ lake | 0 (0.0) | 0 (0.0) | 0 (0.0) | 0 (0.0) |
| • Tanker truck | 0 (0.0) | 0 (0.0) | 0 (0.0) | 0 (0.0) |
| • Bottled water | 0 (0.0) | 0 (0.0) | 0 (0.0) | 0 (0.0) |
| • Rain water | 0 (0.0) | 0 (0.0) | 0 (0.0) | 0 (0.0) |
| • Cart with small tank | 0 (0.0) | 0 (0.0) | 0 (0.0) | 0 (0.0) |
| The principal type of toilet facility used by the household members is: |  |  |  |  |
| • Own (exclusive) flush | 0 (0.0) | 0 (0.0) | 0 (0.0) | 0 (0.0) |
| • Shared flush toilet | 0 (0.0) | 0 (0.0) | 0 (0.0) | 0 (0.0) |
| • Ventilated latrine | 6 (19.4) | 8 (25) | 6 (18.8) | 20 (21.1) |
| • Pit latrine with slab | 9 (29) | 13 (40.6) | 11 (34.4) | 33 (34.7) |
| • Pit latrine without slab/ open pit | 16 (51.6) | 11 (34.4) | 15 (46.9) | 42 (44.2) |
| • Bush or field | 0 (0.0) | 0 (0.0) | 0 (0.0) | 0 (0.0) |
| The number of households using this toilet facility (mean; SD) | 2.55 (1.43) | 2.56 (2.06) | 2.75(2.49) | 2.62 (2.03) |
| The principal type of flooring of the house: |  |  |  |  |
| • Tiles/cement/vinyl | 18 (58.1) | 20 (62.5) | 21 (65.6) | 59 (62.1) |
| • Wood/planks/broken bricks | 1 (3.23) | 0 (0.0) | 0 (0.0) | 1 (1.05) |
| • Dirt/sand/dung | 12 (38.7) | 12 (37.5) | 11 (34.4) | 35 (36.8) |
| The principal type of roofing of the house: |  |  |  |  |
| • Cement | 0 (0.0) | 0 (0.0) | 0 (0.0) | 0 (0.0) |
| • Wood planks/cardboard | 0 (0.0) | 0 (0.0) | 0 (0.0) | 0 (0.0) |
| • Iron and tiles | 2 (6.45) | 0 (0.0) | 1 (3.13) | 3 (3.16) |
| • Iron sheets | 23 (74.2) | 21 (65.6) | 23 (71.9) | 67 (70.5) |
| • Natural materials | 6 (19.4) | 11 (34.4) | 8 (25) | 25 (26.3) |
| A member of the household owns a bank account: | 8 (25.8) | 8 (25) | 7 (21.9) | 23 (24.2) |
| The usual daily income for the family is:  (1 Malawian kwacha (MWK) = 0.0014 United States Dollar) |  |  |  |  |
| • <500 MWK | 3 (9.68) | 2 (6.25) | 1 (3.13) | 6 (6.32) |
| • 500 - 1000 MWK | 23 (74.2) | 19 (59.4) | 23 (71.9) | 65 (68.4) |
| • >1000 MWK | 5 (16.1) | 11 (34.4) | 8 (25) | 24 (25.3) |
| In the past 12 months, the inside walls of the house have been treated against mosquitoes | 2 (6.45) | 3 (9.38) | 1 (3.13) | 6 (6.32) |
| The number of mosquito nets used for sleeping in the household (mean; SD) | 2(1.24) | 2.41(1.88) | 2.13(1.84) | 2.18(1.68) |
| Type of fuel mainly used for cooking: |  |  |  |  |
| • Electricity | 0 (0.0) | 1 (3.13) | 0 (0.0) | 1 (1.05) |
| • Charcoal | 22 (71) | 15 (46.9) | 20 (62.5) | 57 (60) |
| • Wood | 9 (29) | 16 (50) | 12 (37.5) | 37 (38.9) |
| • Natural gas | 0 (0.0) | 0 (0.0) | 0 (0.0) | 0 (0.0) |
| • Biogas | 0 (0.0) | 0 (0.0) | 0 (0.0) | 0 (0.0) |
| • Kerosene | 0 (0.0) | 0 (0.0) | 0 (0.0) | 0 (0.0) |
| • Straw/shrubs/grass | 0 (0.0) | 0 (0.0) | 0 (0.0) | 0 (0.0) |
| Type of stove/fire used for cooking: |  |  |  |  |
| • Open fire | 29 (93.5) | 31 (96.9) | 30 (93.8) | 90 (94.7) |
| • Stove without chimney/ flute | 2 (6.45) | 1 (3.13) | 2 (6.25) | 5 (5.26) |
| • Stove with chimney/ flute | 0 (0.0) | 0 (0.0) | 0 (0.0) | 0 (0.0) |
| Location where cooking is usually done: |  |  |  |  |
| • In the house | 8 (25.8) | 7 (21.9) | 12 (37.5) | 27 (28.4) |
| • In a separate building | 4 (12.9) | 7 (21.9) | 4 (12.5) | 15 (15.8) |
| • Outside | 19 (61.3) | 18 (56.3) | 16 (50) | 53 (55.8) |
| The highest education level of the main care giver is: |  |  |  |  |
| • No education | 3 (9.68) | 2 (6.25) | 1 (3.13) | 6 (6.32) |
| • some primary education | 17 (54.8) | 12 (37.5) | 21 (65.6) | 50 (52.6) |
| • Completed primary education | 3 (9.68) | 5 (15.6) | 2 (6.25) | 10 (10.5) |
| • some secondary education | 6 (19.4) | 11 (34.4) | 5 (15.6) | 22 (23.2) |
| • Completed secondary education | 2 (6.45) | 2 (6.25) | 3 (9.38) | 7 (7.37) |
| • More than secondary education | 0 (0.0) | 0 (0.0) | 0 (0.0) | 0 (0.0) |

Supplementary Table S3 – **Baseline characteristics according to presence of oedema**

| **Variable (N, mean, ± SD)** | **Without Oedema (N=57)** | **With Oedema (N=38)** | **P-value** |
| --- | --- | --- | --- |
| **Demographic** |  |  |  |
| Male (%) | 26/57 (46.7 %) | 20/38 (52.7 %) | 0.50 |
| Age in months | 57, 12.8 ± 4.8 | 38, 17.9 ± 5.4 | <0.001 |
| **Anthropometry** |  |  |  |
| Mid-Upper-Arm-Circumference (cm) | 57, 10.6 ± 1.2 | 38, 14.3 ± 14.2 | <0.001 |
| **Laboratory** |  |  |  |
| Faecal calprotectin (μg/mg) | 48, 660.8 ± 1040.7 | 30, 496.8 ± 545.2 | 0.885 |
| Faecal α_1_-antitrypsin (mg/dL) | 49, 8.4 ± 24.6 | 29, 4.7 ± 5.9 | 0.842 |
| Plasma IgG anti-endotoxin antibodies (GMU/ml) | 34, 94.3 ± 101.9 | 27, 96 ± 144.7 | 0.689 |
| Plasma intestinal fatty acid binding protein (pg/ml) | 34, 4310.6 ± 2652.2 | 26, 4862.8 ± 3150.9 | 0.464 |
| Platelets (x10^9^/L) | 33, 445.8 ± 290.8 | 26, 475.3 ± 234.8 | 0.676 |
| Plasma C-reactive protein (mg/L) | 33, 28.2 ± 50 | 27, 18 ± 21.8 | 0.768 |
| Plasma α_1_-acid glycoprotein (μg/ml) | 34, 2864.4 ± 993.3 | 27, 3403.9 ± 780.2 | 0.024 |
| Insulin-like growth factor-1 (ng/ml) | 22, 51.9 ± 27.5 | 15, 46.7 ± 27.4 | 0.687 |
| Insulin-like growth factor binding protein 3 (ng/ml) | 34, 785.3 ± 354.5 | 26, 888.2 ± 349 | 0.267 |
| Haemoglobin (g/dL) | 33, 9.6 ± 1.1 | 26, 9.2 ± 1.4 | 0.198 |
| White cell count (x10^9^/L) | 33, 12.5 ± 5 | 26, 12.3 ± 5.2 | 0.906 |

Data was analysed using a one-way ANOVA, and variables which were not normally distributed were logarithmically transformed to detect any overall differences in group means according to oedema status

Supplementary Table S4 – **Baseline characteristics according to HIV status**

| **Variable (N, mean, ± SD)** | **HIV Negative (N=60)** | **HIV Positive (N=34)** | **P-value** |
| --- | --- | --- | --- |
| **Demographic** |  |  |  |
| Male (%) | 28 (29.8 %) | 18 (19.1 %) | 0.56 |
| Age in months | 60, 14.9 ± 5.8 | 34, 14.9 ± 5.3 | 0.805 |
| **Clinical** |  |  |  |
| Mid-Upper-Arm-Circumference (cm), | 59, 11.5 ± 1.3 | 34, 10.6 ± 1.4 | 0.002 |
| Weight-for-length Z-score^1^ | 32, -3.5 ± 1.2 | 24, -3.9 ± 1.2 | 0.254 |
| With Oedema (%) | 28 (29.8 %) | 10 (10.6 %) | 0.10 |
| **Laboratory** |  |  |  |
| Faecal calprotectin (μg/mg stool) | 49, 423.5 ± 712.6 | 29, 892.1 ± 1063 | 0.005 |
| Faecal $\alpha_{1}$1-antitrypsin (mg/dL) | 50, 4.6 ± 5.2 | 28, 11.3 ± 32.3 | 0.368 |
| Plasma IgG anti-endotoxin antibodies (GMU/ml) | 41, 84.5 ± 112.9 | 20, 116.8 ± 138.3 | 0.090 |
| Plasma Intestinal fatty acid binding protein (pg/ml) | 40, 4760 ± 2991.7 | 20, 4129.7 ± 2620.5 | 0.427 |
| Platelets (x10^9^/L) | 39, 493.9 ± 259.8 | 20, 390.3 ± 270.5 | 0.158 |
| Plasma C-reactive protein (mg/L) | 40, 16.2 ± 23.6 | 20, 38.4 ± 58.9 | 0.008 |
| Plasma $\alpha_{1}$-acid glycoprotein (μg/ml) | 41, 2945.4 ± 941.2 | 20, 3426.7 ± 865 | 0.059 |
| Insulin-like growth factor -1 (ng/ml) | 29, 49.2 ± 25.5 | 8, 51.7 ± 34.4 | 0.854 |
| Insulin-like growth factor binding protein 3 (ng/ml) | 40, 896.5 ± 381.9 | 20, 696.7 ± 243.7 | 0.038 |
| Haemoglobin (g/dL) | 39, 9.8 ± 1.1 | 20, 8.9 ± 1.3 | 0.007 |
| White cell count (x10^9^/L) | 39, 13 ± 4.7 | 20, 11.3 ± 5.4 | 0.207 |

Data was analysed using a one-way ANOVA, and variables which were not normally distributed were logarithmically transformed to detect any overall differences in group means according to HIV status. ^1^In children without oedema

Supplementary Table S5– **Biomarkers of systemic and intestinal inflammation and mucosal integrity**

|  |  | **All children** | | **Intervention arms** | | | **Intervention vs. standard** | |
| --- | --- | --- | --- | --- | --- | --- | --- | --- |
| **Variable** | **Day** | **Total** n  mean (SD) | **P value^1^** | **Standard**  n  mean (SD) | **Elemental**  n  mean (SD) | **Polymeric**  n  mean (SD) | **Elemental vs. Standard**  P value^2^ | **Polymeric vs Standard**  P value^2^ |
| **Intestinal inflammation** | | | | | | | | |
| Faecal calprotectin (μg/mg; normal <50) | 0 | 71  547 (744) | 0.31 | 23  555 (732) | 25 646 (797) | 23 433 (711) | 0.68 | 0.57 |
|  | 14 | 67 697 (735) |  | 23  538 (643) | 19  803 (788) | 25 763 (777) | 0.24 | 0.28 |
| **Mucosal integrity** | | | | | | | | |
| Faecal α_1_-antitrypsin (mg/dl; normal <26.8) | 0 | 64  25.4 (31.1) | 0.0046 | 23  26.9 (38) | 21  18.4 (12.7) | 20 31 (35.8) | 0.32 | 0.72 |
|  | 14 | 58 46.9 (44.2) |  | 19  30.8 (33.6) | 19  45.3 (37.2) | 20  63.8 (54.1) | 0.22 | 0.028 |
| Plasma IgG anti-endotoxin antibodies (GMU/ml; normal: none detected | 0 | 46  95.6 (128.5) | 0.71 | 15  73.4 (53.6) | 16  151.6 (199.4) | 15  57.9 (45) | 0.15 | 0.4 |
|  | 14 | 45  87.2 (131.7) |  | 17  112.7 (202.6) | 12  63.1(40.4) | 16  78.2 (68.1) | 0.34 | 0.51 |
| Plasma Intestinal fatty acid binding protein (pg/ml; normal 389-2129) | 0 | 44  4474 (2845) | 0.64 | 15  3488 (1970) | 14  4664 (2453) | 15  5284 (3688) | 0.16 | 0.11 |
|  | 14 | 45  4220 (2316) |  | 16  4748 (2735) | 13  4235 (2256) | 16  3682 (1890) | 0.59 | 0.21 |
| **Systemic inflammation** | | | | | | | | |
| Platelets (x10^9^/L blood;  normal 150-450) | 0 | 46  473.9(272.2) | 0.003 | 17  463.8 (302.2) | 13  466.2 (337.5) | 16  490.9 (182.6) | 0.98 | 0.76 |
|  | 14 | 46  593.8(296.5) |  | 16  599.4 (266) | 14  646.0 (300.6) | 16  542.4 (330.5) | 0.66) | 0.59 |
| Plasma C-reactive protein (mg/L; normal 0.104 – 4.19) | 0 | 44  26.4 (44.6) | 0.07 | 14  19.7 (17.8) | 15  21.7 (25.3) | 15  37.3 (70.5) | 0.81 | 0.36 |
|  | 14 | 45  12.2 (28.1) |  | 16  13.1 (29.8) | 13  6.8 (12.7) | 16  15.7(35.6) | 0.46 | 0.82 |
| Plasma α_1_-acid glycoprotein (μg/ml; normal 322-1143) | 0 | 45  3256 (909) | <0.0001 | 14  3315 (855) | 16  3292 (950) | 15  3164 (966) | 0.94 | 0.66 |
|  | 14 | 43  1969 (1001) |  | 15  2530 (1036) | 12  2086 (945) | 16  1356 (650) | 0.26 | 0.0007 |
| **Growth factors** | | | | | | | | |
| Insulin-like growth factor-1 (ng/ml; normal 40-258) | 0 | 27  48 (27) | <0.0001 | 8  43.8 (24.1) | 7  62.1 (30.4) | 12  42.5 (25.9) | 0.22 | 0.91 |
|  | 14 | 29  112.7 (67.4) |  | 9  77.1 (57) | 7  162.8 (83.1) | 13  110.3 (50.7) | 0.028 | 0.17 |
| Insulin-like growth factor binding protein 3 (ng/ml; normal 853-3778) | 0 | 44  827 (337) | <0.0001 | 15  826 (345) | 14  830.8(263) | 15  825 (408) | 0.97 | 1.0 |
|  | 14 | 45  1434 (586) |  | 16  1328 (657) | 13  1427 (3589) | 16  1545 (669) | 0.61 | 0.36 |
| **Anthropometry** | | | | | | | | |
| Mid-upper arm circumference | 0 | 93 11.2 (1.3) | <0.0001 | 30 10.9 (1.4) | 31 11.2 (1.6) | 32 11.4 (0.9) | 0.39 | 0.07 |
|  | 14 | 93 11.8 (1.5) |  | 30 11.4 (1.4) | 31 11.9 (1.7) | 32 12.2 (1.1) | 0.19 | 0.01 |
| Weight | 0 | 91 6.3 (1.5) | <0.0001 | 30 6.5 (1.7) | 30 6.1 (1.8) | 31 6.4 (1.1) | 0.41 | 0.83 |
|  | 14 | 91 6.9 (1.6) |  | 30 7.0 (1.7) | 30 6.7 (1.8) | 31 7.0 (1.3) | 0.61 | 0.78 |
| Weight-for-length Z-score | 0 | 91 -3.2 (1.4) | <0.0001 | 30 -3.4 (1.5) | 30 -3.0 (1.6) | 31 -3.2 (1.2) | 0.30 | 0.47 |
|  | 14 | 91 -2.3 (1.5) |  | 30 -2.6 (1.5) | 30 -2.1 (1.8) | 31 -2.2 (1.2) | 0.19 | 0.27 |

1. Data was analysed using a paired t-test to detect any overall difference between day 0 (up to day 3) and day 14 (± 3 days), and was logarithmically transformed for variables which were not normally distributed. Where the data was still not normal after this transformation a Wilcoxon signed-rank test was performed as an alternative.
2. Independent t-test comparing each intervention treatment group to the standard

Supplementary Table S6 – **Feeding and clinical symptoms according to intervention arm**

| **Variable^1^** | **Standard**  **(N=31)** | **Elemental**  **(N=32)** | **Polymeric**  **(N=32)** | **Elemental vs. standard**  **P value^2^** | **Polymeric vs. standard**  **P value^2^** |
| --- | --- | --- | --- | --- | --- |
| **Feeding** | | | | | |
| - Required nasogastric tube feeds | 6 (19.4) | 15 (46.9) | 11 (34.4) | 0.021 | 0.18 |
| - Required F75 | 8 (25.8) | 6 (18.8) | 0 (0) | 0.50 | 0.002 |
| No. days completed feed: mean (SD) | 12.0 (4.1) | 11.0 (4.3) | 12.1 (3.5) | 0.32 | 0.98 |
| **Symptoms and signs** | | | | | |
| Stool frequency/day | 3.6 (1.93) | 3.9 (2.0) | 3.4 (1.9) | 0.047 | 0.13 |
| Children who experienced between day 0 and 14 | | | | | |
| - Vomiting | 10 (32.3) | 19 (59.4) | 19 (59.4) | 0.031 | 0.031 |
| - Loose stools | 22 (71) | 26 (81.3) | 26 (81.3) | 0.34 | 0.34 |
| - Watery stools | 5 (16.1) | 9 (28.1) | 7 (21.9) | 0.25 | 0.56 |
| - Mucus in stools | 0 (0) | 2 (6.3) | 0 (0) | 0.49 | - |
| - Blood in stools | 1 (3.23) | 0 (0) | 2 (6.25) | 0.49 | 1.0 |
| On day 14, children with | | | |  |  |
| - Loose/watery stools | 6 (26.1) | 5 (19.2) | 6 (18.8) | 0.97 | 1.0 |
| - Any oedema | 2 (6.5) | 3 (9.4) | 1 (3.1) | - | - |

^1^Data are n (%) unless shown otherwise. ^2^ Data was analysed using a Chi-Square test for n (%) data, a t-test for stool frequency/day and Generalized Linear Analysis for number of days completed feed with Poisson distribution and a link log.

Supplementary Table S7 – **Severe adverse events according to MedDRA preferred term, intervention group and outcome**

|  | **Total** | **Standard** | **Elemental** | **Polymeric** |
| --- | --- | --- | --- | --- |
|  |  | **N=31** | **N=32** | **N=32** |
| **Number (%) of children** | **25** | 9^2^ (29.0) | 9^2^ (28.1) | 7 (21.9) |
| MedDRA preferred term | | | | |
| ·      Gastroenteritis | 13 | 4 | 8 | 1 |
| ·      Dehydration | 11 | 5 | 5 | 1 |
| ·      Sepsis | 6 | - | 3 | 3 |
| ·      Metabolic acidosis | 3 | - | 2 | 1 |
| ·      Pulmonary tuberculosis^1^ | 3 | 1 | - | 2 |
| ·      Pneumonia | 1 | 1 | - | - |
| ·      Septic shock | 1 | - | 1 | - |
| ·      Hypoglycaemia | 1 | - | - | 1 |
| ·      Urticaria drug-induced | 1 | 1 | - | - |
| ·      Hypovolemic shock | 1 | 1 | - | - |
| ·      Acute kidney injury | 1 | - | 1 | - |
| ·      Unknown^3^ | 1 | - | - | 1 |
| Total number SAEs | 43 | 13 | 20 | 10 |
| Total number deaths | 7 | 2 | 2 | 3 |

A total of 43 SAEs occurred in 25 children. ^1^Treatment for tuberculosis was on-going in these 3 cases at the end of the study. ^2^Two patients (one in the control group and one in the elemental group) had an SAE onset within the study period but died one day after the 14th day. ^3^20-month-old boy, HIV positive, malaria slide negative, admitted with oedematous SAM, gastroenteritis, oral thrush and septicaemia. He was reported to be well on review on the third study day but then died suddenly and the cause was unclear. The results of an initial blood culture were not available.
